# Effects and mechanisms of vitamin A and vitamin E on the levels of serum leptin and other related cytokines in rats with rheumatoid arthritis

**DOI:** 10.3892/etm.2014.1777

**Published:** 2014-06-11

**Authors:** RI-BO XIONG, QING LI, WEI-REN WAN, JIN-QIANG GUO, BING-DE LUO, LU GAN

**Affiliations:** 1 Department of Child and Adolescent Health, School of Public Health and Tropical Medicine, Southern Medical University, Guangzhou, Guangdong 510515, P.R. China; 2 Department of Nutrition of Nanfang Hospital, Southern Medical University, Guangzhou, Guangdong 510515, P.R. China

**Keywords:** rheumatoid arthritis, leptin, vitamin A, vitamin E, p-STAT1, p-STAT3

## Abstract

Leptin has been identified as an important cytokine in the inflammatory networks of rheumatoid arthritis (RA). Higher serum leptin levels may accelerate the development of RA. This study aimed to examine the effects of vitamin A (VitA) and vitamin E (VitE) on the levels of leptin and other related experimental and clinical indices, and to explore the mechanisms of these effects through the Janus kinase/signal transducer and activator of transcription (STAT) signal transduction pathway in rats with collagen-induced arthritis (CIA). CIA model rats were established by the intradermal injection of bovine type II collagen emulsified in incomplete Freund’s adjuvant, followed by a booster intradermal injection. Four weeks later, the CIA model rats were treated with 42.86 μg retinol equivalents/kg body weight (b.w.) VitA or 200 mg/kg b.w. VitE for four weeks. The levels of leptin, tumor necrosis factor-α (TNF-α), interleukin (IL)-6, IL-10, IL-4, C-reactive protein (CRP) and rheumatic factor were measured by ELISA using commercial kits, and the erythrocyte sedimentation rate (ESR) was determined. In addition, the expression levels of phosphorylated (p)-STAT1, p-STAT3 and leptin in the synovium were evaluated by western blot analysis. The results indicated that VitA and VitE significantly reduced the levels of leptin, TNF-α, IL-6 and CRP and the ESR and significantly increased the levels of IL-10 compared with those of the model group. Furthermore, significantly reduced p-STAT3 protein expression levels were observed in the VitA and VitE groups. In conclusion, VitA and VitE reduced the levels of serum leptin protein and other cytokines. Furthermore, VitA and VitE also reduced the p-STAT3 protein levels. The present study may provide a novel approach for the treatment of RA.

## Introduction

Rheumatoid arthritis (RA) is an autoimmune inflammatory disease that has been shown to be associated with the destruction of articular cartilage and loss of the function of joints (1). A study demonstrated that complex cytokine networks exist in RA, the biological effects of which are associated with the relative serum concentrations of inflammatory cytokines and their inhibitors. The interactions of cytokines play an important role in inflammation, adhesion, neovascularization and decreased bone density (2).

Leptin is a type of hormone constitutively secreted by adipose tissue with a molecular weight of 16 kDa. It was originally described as a regulator of food intake and energy expenditure (3). A study revealed that leptin plays an important role in autoimmune diseases through proinflammatory functions on T-helper type 1 (Th1) cells (4). Patients with RA in the acute phase exhibit increased serum leptin levels and the leptin concentration in the joint fluid was lower than with that in the serum (5). A positive correlation between the levels of leptin and proinflammatory cytokines, including tumor necrosis factor-α (TNF-α) and interleukin (IL)-6, has been identified (6). However, the association between leptin levels and RA activity levels of has not been fully elucidated. The Janus kinase (JAK)/signal transducer and activator of transcription (STAT) signal transduction pathway is a major mediator of the biological effects of leptin, including proinflammation and immunological regulation. When leptin combines with a leptin receptor, the corresponding molecules are activated and transported to the cell nucleus, which promotes the transcription of target genes that involves two key molecules: Phosphorylated (p)-STAT1 and p-STAT3. Therefore, lowering leptin levels may be an important strategy in the treatment of RA (7).

RA is a chronic disease that requires the intake of drugs, including antirheumatics, non-steroidal anti-inflammatory drugs and biological agents (8). Patients are prone to discontinue treatment due to the side-effects of the drugs. It has been reported that 33–75% of RA patients consider food to play an important role in their severity of their symptoms and 20–50% have tried dietary manipulation in an attempt to relieve suffering (9,10). It has been indicated that vitamins with antioxidant properties may help to treat RA. Antioxidants, including vitamin A (VitA) and vitamin E (VitE), have been demonstrated to manifest inhibitory effects on inflammatory cytokines *in vivo* (11,12). However, there are no studies concerning the effects of VitA and VitE on the leptin levels of rats with RA. Therefore, the present study aimed to examine the effects of VitA and VitE on the levels of leptin and other related experimental and clinical indices in rats with collagen-induced arthritis (CIA) and to explore the possible mechanisms of these effects associated with the signal transduction pathway of leptin.

## Materials and methods

### Animals and treatments

Male Wistar rats (147±15 g) from Southern Medical University Laboratory Animal Co. Ltd. (Guangzhou, China) were used in the experiments. The animal care and study protocols employed were in accordance with the guidelines of the Animal Care and Use Committee of Southern Medical University (Guangzhou, China) and the Organization for Economic Co-operation and Development (13). The rats were housed in cages in a climate-controlled room with a 12-h light-dark cycle. Throughout the study, the animals were allowed access to regular standard rats chow and water *ad libitum*.

After a one-week acclimation period, the animals were administered an intradermal injection (100 μl) of bovine type II collagen emulsified in incomplete Freund’s adjuvant or 0.9% normal saline (the model and control groups, respectively). Two weeks later, the rats were administered a booster intradermal injection. At the end of the fourth week, the arthritis index (14) was applied to evaluate paw swelling. Each paw was graded on a scale of 0–4 as follows: 0, normal, without any macroscopic signs of arthritis; 1, mild, but definite redness and swelling of the ankle or apparent redness and swelling limited to individual digits, regardless of the number of affected digits; 2, moderate redness and swelling of the ankle; 3, redness and swelling of the entire paw including the digits; and 4, maximally inflamed limb with involvement of multiple joints. The four paw scores for each animal were summed. The rats with a score of >6 were used in the following experiments.

The model group was divided into four subgroups: i) VitA [42.86 μg retinol equivalents/kg body weight (b.w.)] (n=6); ii) VitE (200 mg/kg b.w.) (n=6); iii) ibuprofen (50 mg/kg b.w.) (n=6) and iv) untreated model groups (n=6). The rats in the VitA, VitE and ibuprofen groups received intragastric administration of VitA, VitE and ibuprofen, respectively, once daily for four weeks. At the end of the eighth week, all rats were anaesthetized and sacrificed, and blood samples and joint synovium tissue were extracted. The tissue was stored at −80°C until it was used for western blot analysis.

### Determination of serum leptin levels and other related experimental and clinical indices

Serum was isolated from the blood samples by centrifugation at 11.1 × g for 10 min and then maintained at −20°C prior to the following assays. The levels of leptin, TNF-α, IL-6, IL-10, IL-4, C-reactive protein (CRP) and rheumatic factor (RF) were measured by ELISA using commercial kits (R&D Systems, Inc., Minneapolis, MN, USA). The erythrocyte sedimentation rate (ESR) was also determined. ESR was determined by automated erythrocyte sedimentation rate analyzer (ELECTA LAB S.r.l, Via Balzella, Italy).

### Western blot analysis of p-STAT1, p-STAT3 and leptin expression levels

The tissue samples were homogenized in complete radioimmunoprecipitation assay lysis buffer (Santa Cruz Biotechnology, Inc., Dallas, TX, USA). The total protein was quantified with a bicinchoninic acid protein assay kit. All preparations were performed at 4°C. For western blotting, a total of 15 μl of the mixture of protein and sample buffer was loaded per lane and the proteins were electrophoretically separated on an SDS-PAGE gel. The protein bands were transferred to a polyvinylidene fluoride membrane using a Trans-Blot SD Semi-Dry Electrophoretic Transfer cell (Santa Cruz Biotechnology, Inc.). The gels were then incubated with primary antibodies (Santa Cruz Biotechnology, Inc.) against p-STAT1, p-STAT3 and leptin overnight at 4°C. Subsequently, the gels were incubated with secondary antibodies (Santa Cruz Biotechnology, Inc.) for 1 h at room temperature. The films were scanned and analyzed using Quantity One (Bio-Rad Laboratories Inc., Berkeley, CA, USA) to quantify the protein levels. The relative protein levels were counted by comparison with the beta-actin control.

### Statistical analysis

Data are expressed as the mean ± standard deviation. Statistical differences between groups were compared by one-way analysis of variance with SPSS software for statistical analysis, version 16.0 (SPSS, Inc., Chicago, IL, USA). P<0.05 was considered to indicate a statistically significant difference.

## Results

### Effects of VitA and VitE on the levels of leptin

**Figure 1:**
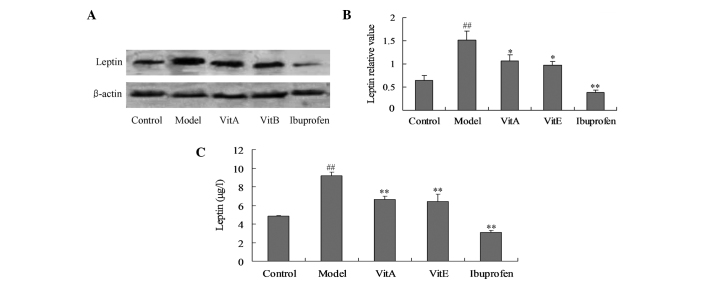
Effects of VitA and VitE on the leptin levels in CIA model rats. (A) Western blot analysis of joint synovium tissue; (B) statistical analysis of the western blotting; and (C) ELISA of the serum leptin levels. Data are presented as the mean ± SD, ^##^P<0.01 vs. the control group; ^*^P<0.05 vs. the model group;^**^P<0.01 vs. the model group. VitA, vitamin A; VitE, vitamin E; CIA, collagen-induced arthritis.

The leptin levels in the joint synovium tissue and serum were detected by western blot analysis and ELISA, respectively. Compared with the those of the control animals, the leptin levels were significantly increased in the untreated model animals, as detected by the western blot analysis and ELISA (Fig. 1; P<0.01). Four-week administration of VitA and VitE significantly reduced the levels of leptin compared with those of the untreated model animals (Fig. 1; P<0.05).

### Effects of VitA and VitE on the levels of serum TNF-α, IL-6, IL-10 and IL-4

Compared with those of the control group, there were significant increases in the serum TNF-α and IL-6 levels and significant reductions in the serum IL-10 and IL-4 levels in the untreated model group (P<0.01). Four weeks of VitA and VitE administration significantly reduced the levels of TNF-α (P<0.05) and IL-6 (P<0.01) compared with those of the untreated model group. In addition, significant increases in the serum IL-10 levels was identified in the VitA and VitE groups compared with those of the untreated model group, but the changes in the serum IL-4 levels were not found to be significant.

### Effects of VitA and VitE on the ESR and the levels of CRP and RF

**Figure 3:**
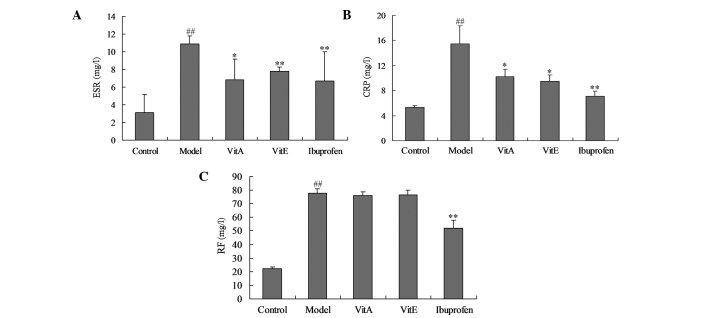
Effects of VitA and VitE on the ESR, and the levels of CRP and RF. Serum (A) ESR, (B) CRP level and (C) RF level in CIA model rats. Data are presented as the mean ± SD, ^##^P<0.01 vs. the control group; ^*^P<0.05 vs. the model group; ^**^P<0.01 vs. the model group. ESR, erythrocyte sediment rate; CRP, C-reactive protein; RF, rheumatic factor; VitA, vitamin A; VitE, vitamin E; CIA, collagen-induced arthritis.

ESR, CRP and RF are markers of the disease activity index in RA. Fig. 3 shows the changes in the levels of these markers. Compared with those of the control group, the ESR, and the CRP and RF levels in the untreated model group were significantly increased (P<0.01). Treatment with VitA or VitE was associated with significant reductions in the ESR and CRP levels (P<0.05), but the levels of RF were not found to be significantly reduced compared with those of the untreated model group.

### Effects of VitA and VitE on p-STAT1 and p-STAT3 protein expression levels

**Figure 4:**
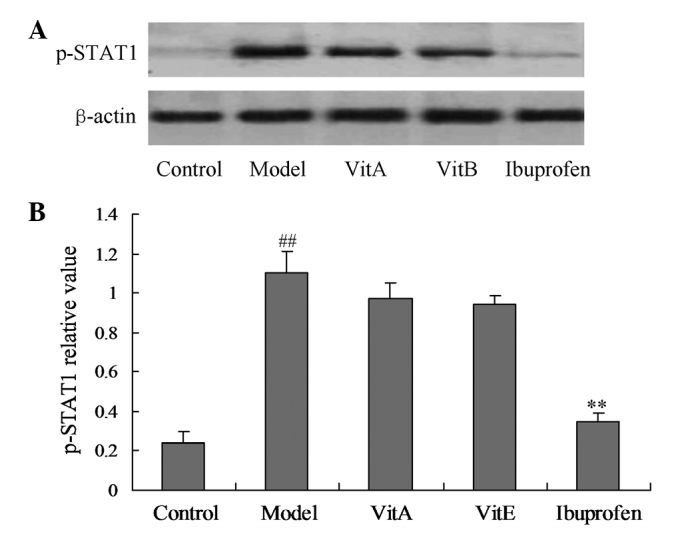
Effects of VitA and VitE on the p-STAT1 protein expression levels in CIA model rats. (A) Western blot analysis of p-STAT1 protein levels. (B) Statistical analysis of the p-STAT1 expression levels. The relative quantification of the p-STAT1 protein levels is expressed as a ratio of the target protein to β-actin. Values were normalized with respect to β-actin and expressed as a percentage of the control. Each value represents the mean ± SD, ^##^P<0.01 vs. the control group; ^**^P<0.01 vs. the model group. p-STAT1, phosphorylated-signal transducer and activator of transcription 1; VitA, vitamin A; VitE, vitamin E; CIA, collagen-induced arthritis.

**Figure 5:**
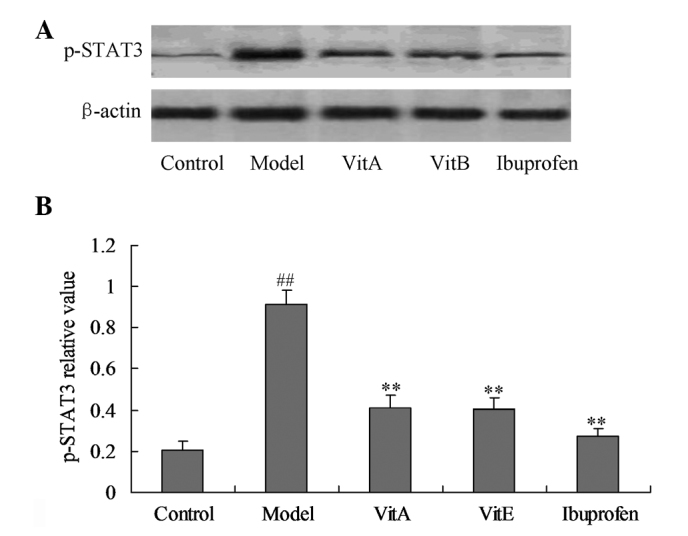
Effects of VitA and VitE on the p-STAT3 protein expression levels in CIA model rats. (A) Western blot analysis of p-STAT3 protein levels. (B) Statistical analysis of the p-STAT3 expression levels. The relative quantification of the p-STAT3 protein levels was expressed as a ratio of the target protein to the β-actin. Values were normalized with respect to β-actin and expressed as a percentage of the control. Each value represents the mean ± SD, ^##^P<0.01 vs. the control group; ^**^P<0.01 vs. the model group. p-STAT3, phosphorylated-signal transducer and activator of transcription 3; VitA, vitamin A; VitE, vitamin E; CIA, collagen-induced arthritis.

p-STAT1 and p-STAT3 are molecules associated with the JAK/STAT signal transduction pathway. Figs. 4 and 5 show the effects of VitA and VitE on the p-STAT1 and p-STAT3 protein expression levels. The model group showed a significant upregulation of the p-STAT1 and p-STAT3 protein expression levels compared with those of the control group (Figs. 4 and 5; P<0.01). The rats treated with VitA or VitE for four weeks showed significant reductions of the p-STAT3 protein expression levels (P<0.01), but the treatment effect was not significant for the p-STAT1 protein expression levels compared with those of the untreated model group.

## Discussion

The present study indicates the role of leptin in the pathogenesis of CIA in rats and the effects of VitA and VitE on cytokine networks and other related indices. The study also examined the effects of VitA and VitE on p-STAT1 and p-STAT3 protein expression levels, which are markers of the signal transduction pathway of leptin.

An unbalanced cytokine network exists in the pathogenesis of RA, which manifests as increased levels of inflammatory cytokines and reduced levels of anti-inflammatory cytokines (15). In the present study, compared with those of the control group, rats with CIA had higher levels of TNF-α and IL-6 and lower levels of IL-4 and IL-10, which is consistent with the results in other studies (16,17). The results of the present study indicated that TNF-α and IL-6 were inflammatory cytokines while IL-4 and IL-10 were anti-inflammatory cytokines in the pathogenesis of RA. The study also observed the alterations in the levels of leptin. A previous study has demonstrated increased serum leptin levels in patients with RA (5). In the present study, increased leptin levels existed in the rats with CIA, which indicates that leptin plays an important role in RA and is identifiable as an inflammatory cytokine. The possible mechanisms involving leptin include two scenarios. One is that leptin increases the secretion of inflammatory cytokines by the induction of Th1-cell differentiation, and the other is that leptin suppresses the apoptosis of senile cells and this results in the deterrence of autoantigen clearance (18).

RA is a chronic disease that requires long-term intake of drugs, including antirheumatics and non-steroidal anti-inflammatory drugs. Patients with RA are prone to drop out of drug treatment due to the adverse effects. In RA, free radicals are associated with joint inflammation and damage. Antioxidant supplements and diets have long been advocated for the treatment and prevention of RA due to their protective role against free radicals (19). It has been reported that 33–75% of RA patients consider that food plays an important role in the severity of their symptoms and 20–50% have tried dietary manipulation in an attempt to relieve their suffering (9,10). Epidemiological studies have shown that a low intake of dietary antioxidants is associated with the incidence of RA (20,21). Despite the fact that vitamins with antioxidant properties have been demonstrated to be beneficial to RA in a cellular study, there are contradicting results concerning the effects of antioxidant vitamins on the development of RA in animal and clinical studies (22–24). In the present study, the effects of VitA and VitE on the inflammatory cytokine networks were observed in CIA model rats. When treated with VitA and VitE for four weeks, the levels of leptin, TNF-α and IL-6 were significantly reduced while the levels of IL-10 were significantly increased. This suggests that VitA and VitE suppress the inflammatory reaction in RA by increasing the levels of anti-inflammatory cytokines and reducing the levels of inflammatory cytokines. Due to the complicated cytokine networks in RA, cytokines interact by several signal transduction pathways. Therefore, the stimulative effects of VitA and VitE on IL-4 levels may be counteracted by the interaction between cytokines in RA. This explains the observation that the levels of IL-4 were not increased following treatment with VitA and VitE. Thus, the interactions of cytokines in RA require further investigation.

The present study also examined the effects of VitA and VitE on the ESR, and the levels of CRP and RF, which reflect the levels of disease activity in RA. An elevated ESR indicates the body is in a pathological status. Thus, the ESR is widely used in the monitoring of the levels of disease activity in patients with various ailments, including infection and inflammation (25). CRP is a reactive protein in the acute phase response, which is observed at increased levels in a number of ailments, including tissue damage, myocardial infarction and malignant tumors (26). RF is one of most widely used indicators in RA and although it has a low specificity, a study has suggested that RF is one of the most potent factors in the prognosis of RA due to its intimate association with joint damage (27). The results of the present study show that VitA and VitE reduce the ESR and CRP levels, the indicators of disease activity. The evidence that the levels of RF are not altered implies that RF is not a specific index of CIA in rats.

The present study adds a novel dimension to the protective effects of VitA and VitE on CIA rats by the assessment of the levels of p-STAT1 and p-STAT3 proteins, two key molecules of the JAK/STAT signal transduction pathway. The JAK/STAT signal transduction pathway is a major mediator of the biological effects of leptin, including cell proliferation and differentiation, immunological regulation and inflammation in RA. Once leptin is combined with a leptin receptor, the corresponding molecules are activated and transported to the cell nucleus, which promotes the transcription of the target genes p-STAT1 and p-STAT3 (7). In the present study, compared with those of the control group, the expression levels of p-STAT1 and p-STAT3 were significantly increased in the untreated model group, which suggests they are involved in the pathogenesis of RA. In the CIA model rats treated with VitA and VitE, the levels of p-STAT3 were significantly reduced, which is consistent with a previous study that showed p-STAT3 expression levels are upregulated in zymosan-induced arthritis (28). However, this phenomenon was not observed for p-STAT1 (29,30). In the present study, the difference in the effects of VitA and VitE between p-STAT1 and p-STAT3 suggests that the two molecules have disparate functions in RA. A clinical study involving 30 patients with RA identified that upregulation of p-STAT1 expression levels increased the inflammation in the synovium of joints through activation of the expression of related genes (29). Another cellular study showed that increased p-STAT1 expression levels promote apoptosis of synovium cells and suppress the inflammatory response (30). In the present study, p-STAT1 expression levels were not significantly reduced, which indicates p-STAT1 may play a protective role by suppressing the proliferation of synovial cells. The mechanism by which p-STAT3 expression levels alone are reduced requires further study.

A limitation of the present study is that the protective effects of VitA and VitE observed in the animal model of RA may not be similar to those observed clinically. The interactions of leptin and other cytokines require further study. These results may have implications for the rational development of antioxidant vitamins for the treatment of RA.

In conclusion, VitA and VitE reduced the levels of serum leptin protein and other cytokines in a murine model of RA. Furthermore, VitA and VitE also reduced the levels of p-STAT3 protein. The present study may provide a novel approach for the treatment of RA.

## Figures and Tables

**Figure 2:**
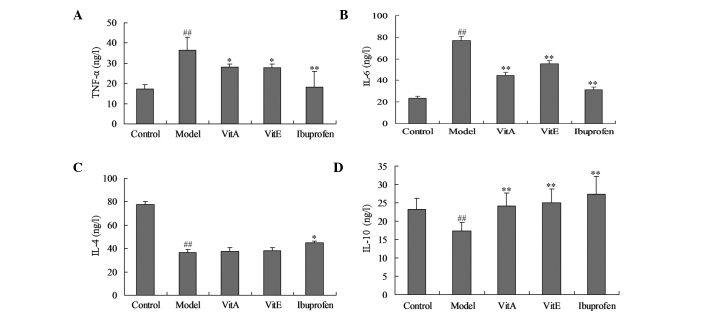
Effects of VitA and VitE on the levels of serum cytokines in CIA model rats. Serum (A) TNF-α, (B) IL-6, (C) IL-4 and (D) IL-10 levels. Data are presented as the mean ± SD, ^##^P<0.01 vs. the control group; ^*^P<0.05 vs. the model group; ^**^P<0.01 vs. the model group. TNF-α, tumor necrosis factor-α; IL, interleukin; VitA, vitamin A; VitE, vitamin E; CIA, collagen-induced arthritis.
